# Liver damage in schistosomiasis is reduced by adipose tissue-derived stem cell therapy after praziquantel treatment

**DOI:** 10.1371/journal.pntd.0008635

**Published:** 2020-08-27

**Authors:** Vitor Hugo Simões Miranda, Talita Rocha Gomes, Dirli Emerick Eller, Lorena de Cássia Neres Ferraz, Ana Thereza Chaves, Kelly Alves Bicalho, Carlos Eduardo Calzavara Silva, Alexander Birbrair, Marcelo Antônio Pascoal Xavier, Alfredo Miranda de Goes, Rodrigo Corrêa-Oliveira, Érica Alessandra Rocha Alves, Adriana Bozzi

**Affiliations:** 1 Instituto René Rachou, Fiocruz Minas, Belo Horizonte, Minas Gerais, Brazil; 2 Departamento de Morfologia, Universidade Federal de Minas Gerais (UFMG), Belo Horizonte, Minas Gerais, Brazil; 3 Faculdade de Medicina, UFMG, Belo Horizonte, Minas Gerais, Brazil; 4 Departamento de Patologia, ICB, UFMG, Belo Horizonte, Minas Gerais, Brazil; 5 Departamento de Bioquímica e Imunologia, ICB, UFMG, Belo Horizonte, Minas Gerais, Brazil; 6 Departamento de Ciências Biológicas, Universidade Estadual de Santa Cruz, Ilhéus, Bahia, Brazil; Centers for Disease Control and Prevention, UNITED STATES

## Abstract

**Background:**

In view of the potential immunosuppressive and regenerative properties of mesenchymal stem cells (MSC), we investigated whether transplantation of adipose tissue-derived stem cells (ASC) could be used to control the granulomatous reaction in the liver of mice infected with *Schistosoma mansoni* after Praziquantel (PZQ) treatment.

**Methodology/Prinicpal findings:**

C57BL/6 mice infected with *S*. *mansoni* were treated with PZQ and transplanted intravenously with ASC from uninfected mice. Liver morpho-physiological and immunological analyses were performed. The combined PZQ/ASC therapy significantly reduced the volume of hepatic granulomas, as well as liver damage as measured by ALT levels. We also observed that ASC accelerated the progression of the granulomatous inflammation to the advanced/curative phase. The faster healing interfered with the expression of CD28 and CTLA-4 molecules in CD4^+^ T lymphocytes, and the levels of IL-10 and IL-17 cytokines, mainly in the livers of PZQ/ASC-treated mice.

**Conclusions:**

Our results show that ASC therapy after PZQ treatment results in smaller granulomas with little tissue damage, suggesting the potential of ASC for the development of novel therapeutic approaches to minimize hepatic lesions as well as a granulomatous reaction following *S*. *mansoni* infection. Further studies using the chronic model of schistosomiasis are required to corroborate the therapeutic use of ASC for schistosomiasis.

## Author summary

Schistosomiasis is the second most prevalent parasitic disease in the world and is caused by the *Schistosoma* trematode. This disease is characterized by a granulomatous reaction around parasite eggs trapped in the tissues. The liver is one of the most affected organs and can develop severe fibrosis. Praziquantel (PZQ) is the treatment for schistosomiasis and kills the adult the worm; however, inflammation still persists around the eggs in the tissues. Mesenchymal stem cells (MSC) have been extensively studied as an alternative therapy to repair tissues and to stop inflammation due to their potential to differentiate in several cells types (bone, cartilage, fat, tendon, muscle, and marrow stroma), and to interfere with immune responses. This scenario has motivated the authors to investigate the use of MSC extract from adipose tissue (ASC) associated with PZQ to treat schistosomiasis. Briefly, mice were treated with PZQ followed by ASC injection showing significant reduction of the granulomas and normal levels of the enzyme alanine aminotransferase, an indicator of liver damage. These results suggest that ASC has the potential to be used as a novel therapeutic approach to control inflammation following infection by *S*. *mansoni* or liver disorders. Although the findings are promising, further studies using the chronic model of schistosomiasis are required to confirm using ASC for schistosomiasis therapy.

## Introduction

Schistosomiasis is a helminthic infection that is associated with severe morbidity and has a significant socioeconomic impact on the affected populations. Over 240 million people are estimated to be infected and 700 million are at risk of infection globally [1]. Schistosomiasis is caused by 6 species of trematodes of the *Schistosoma* genus, however, the predominant causes of the disease are either *S*. *mansoni* or *S*. *hematobium* [2]. *S*. *mansoni* adult worms colonize human blood vessels [3] and produce eggs that become trapped in the tissues [4–6]. These eggs trigger the inflammation that is followed by the development of granulomas, mainly in the liver and intestine, which may result in severe fibrosis and portal hypertension [7, 8]. During *S*. *mansoni* infection, the granulomatous inflammation is dependent on CD4^+^ T lymphocytes, macrophages [9], eosinophils, and collagen fibers [10]. The chronic inflammation, induced mainly by soluble egg antigens (SEA) in the tissues, primed by Omega-1, a glycoprotein from SEA [11–13], leads to cellular production of regulatory Th2 cytokines resulting in the modulation of Th1 response and reduction of the granuloma size in most individuals. This immunological process is essential for controlling morbidity. Our group and others have already shown that IL-10 is the main factor in generating conditions for the host protective homeostatic functions in schistosomiasis [6, 14–17]. However, a significant number of individuals lack the ability to control the inflammatory response and, as a consequence, develop the severe form of the disease.

Interventions to control schistosomiasis have varied over time. Due to the low cost, safety and high efficacy, PZQ has been used as the gold-standard treatment against schistosomiasis, and its mass administration has become the mainstay of national control programs [18]. Currently, there is no specific treatment to control the granulomatous reaction and hepatosplenic clinical form of the disease, which might continue for several months after successful treatment with PZQ [19].

Mesenchymal stem cells (MSC) [20, 21] have gained significant attention as a promising therapy for several diseases. This scenario is mainly due to their plasticity that allows the use of MSC as a regenerative agent, as well as their immunomodulatory activity [22]. MSC can be easily obtained from many adult tissues, including bone marrow [23], umbilical cord, and adipose tissues [24, 25]. Under appropriate conditions, MSC can differentiate into cells of the mesodermal lineage, such as adipocytes, osteocytes, and chondrocytes, as well as other embryonic cell types [22]. Furthermore, MSC can interact with cells of both innate and adaptive immune responses, leading to the down-modulation of several effector functions. The immunosuppressive properties of MSC, including anti-proliferative and anti-inflammatory effects, are mainly a result of their production of high levels of gamma interferon (IFN-γ) and tumor necrosis factor-α (TNF-α) [26, 27]. The putative immunosuppressive and regenerative potential of MSC in combination with their proposed low immunogenicity open new avenues to control the inflammation in a variety of diseases [28], including orthopedic injuries, graft *versus* host disease (GVHD), cardiovascular, autoimmune, and liver diseases [29–34]. However, the effect of stem cells on the inflammatory response associated with parasitic diseases has not been well investigated yet. Recently, the effect of stem cells on parasitic diseases such as malaria [35] and Chagas [36–41] was reported. However, only a few preview studies have analyzed the effect of MSC on *Schistosoma* infection [42–46].

Considering the immunosuppressive and regenerative properties of MSC, we have hypothesized that their use as adjunctive therapy would reduce the granulomatous response to *S*. *mansoni* eggs and promote faster patient recovery. Thus, in the present study, we have evaluated a therapy protocol, to test the effect of the administration of ASC after using PZQ to treat C57BL/6 mice infected with *S*. *mansoni*, and have analyzed the impact of this therapy on the granuloma reaction as well as liver damage resulting from infection.

## Materials and methods

### Animals

**Fig 1:**
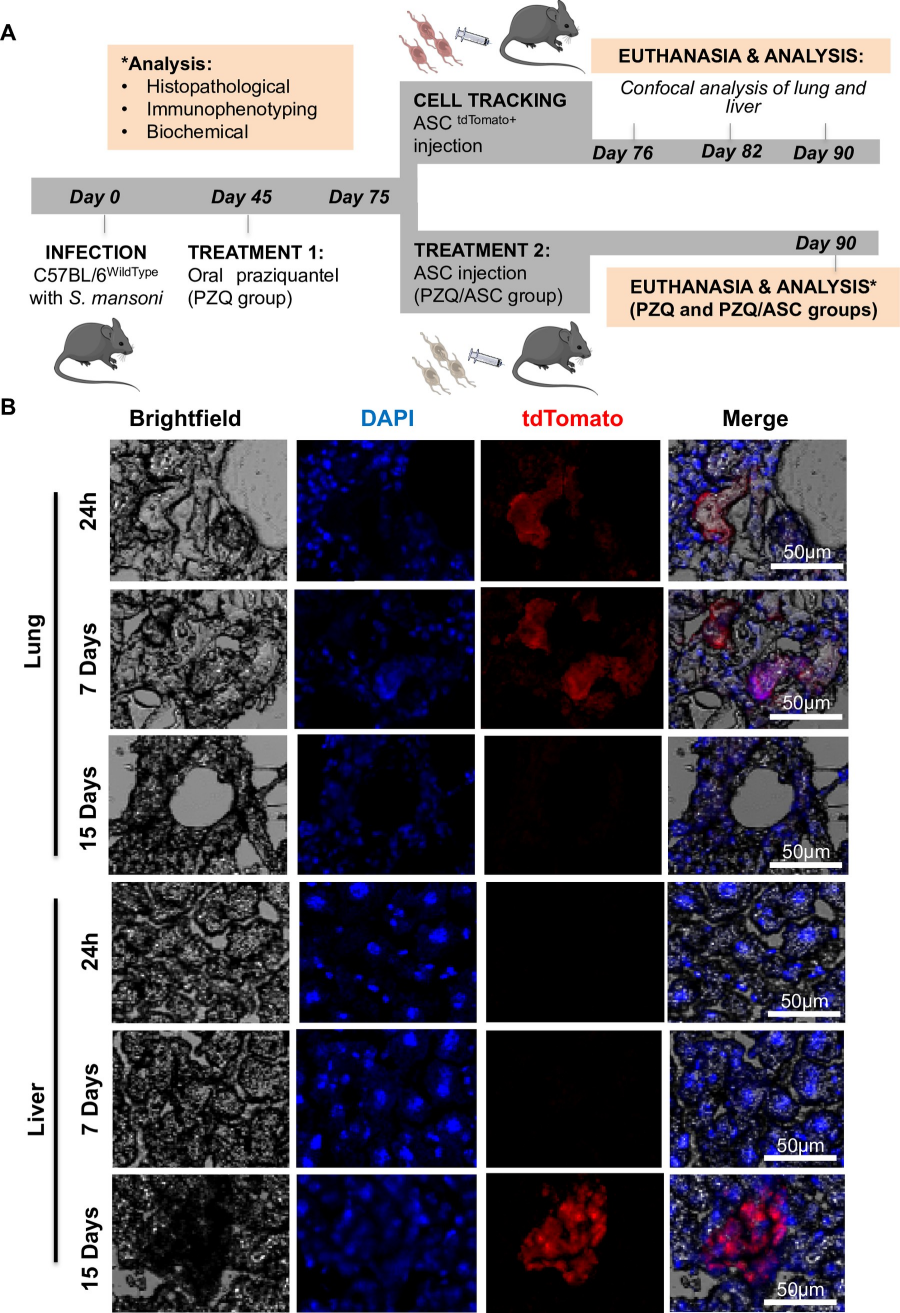
Experimental design and tracking of ASC ^tdTomato+^. Panel A shows the experimental design of the study. C57BL/6 mice were infected with *S*. *mansoni* (Day 0), treated orally with PZQ (Day 45; PZQ group), and then injected with ASC (3x10^5^) (Day 75; PZQ/ASC group) or PBS (PZQ). The mice from PZQ and PZQ/ASC groups were analyzed on Day 90. In parallel, other group treated with PZQ was injected with 3x10^5^ tdTomato-expressing ASC derived from ROSA^mT/mG^ mice. After 1 day, 7 days, and 15 days, the animals were euthanized, and their liver and lungs collected for confocal analysis. Note: an infected not treated group (NT group) was followed until day 90-post infection as a control for the PZQ treatment. Panel B shows representative confocal cell images of the lung and the liver 1, 7 and 15 days after ASC^tdTomato+^ injection. Cryosections were counterstained with DAPI (blue fluorescence). Red fluorescence refers to ASC^tdTomato+^. Images were captured using the Ti-E C2 plus microscope confocal with magnification of 100x, and analyzed using NIS-Elements (Nikon).

The present study has been approved by the Animal Research Ethical Committee at the Fiocruz, Rio de Janeiro (Process number: LW-56/14). The experimental design is shown in the Fig 1A. Six to eight-week-old male C57BL/6 and ROSA^mT/mG^ mice were used for the different experiments.

### ASC extraction and culture

ASC were isolated from 7-week-old C57BL/6 mouse inguinal adipose tissue as described previously [47], washed with phosphate-buffered saline (PBS) 0.15M and enzymatically digested with 0.15% collagenase type II (Life Technologies, California, USA) in Dulbecco’s modified Eagle’s medium (DMEM; Gibco, California, USA) at 37°C for 50 min. Subsequently, the stromal vascular fraction was obtained by centrifugation at 252 x *g* for 10 min, and the pellet was resuspended in the basal medium [DMEM supplemented with 10% fetal bovine serum (Gibco), 60 g/L gentamicin [48], 25 g/L amphotericin B, 100U/mL penicillin, 100 g/mL streptomycin (Gibco)]. The cells were seeded into polystyrene cell culture flasks T75mm^3^ (TTP; Schaffhausen, Switzerland), incubated at 37°C for 24h, and non-adherent cells were removed.

### Viability assay

To analyze the viability of ASC preparations, the MTT reduction test was used. Briefly, cells were seeded into 24 well plates (2x10^5^) and cultured in the basal medium at 37°C in 5% CO_2_ for 24h. Staurosporine (100nM) (Sigma, Missouri, USA) was added in culture for negative control (non-viable cells). Afterwards, ASC were incubated with MTT (Sigma) at 37°C in 5% CO_2_ for 2h, followed by incubation for 12h with 10% sodium dodecyl sulfate (SDS) in 1N HCl to solubilize the formazan product. The absorbance at 595 nm was measured using a microtiter plate reader (Molecular Devices, California, USA). The mean absorbance and standard deviation (SD) were determined in triplicate for each experimental group.

### Phenotypic characterization of ASC

ASC were detached with 0.25% trypsin/EDTA (Sigma), centrifuged for 5 min at 379 x *g*, and the cell pellet suspended in PBS 0.15M. Aliquots of 5x10^5^ cells were incubated for 30 min at 4°C with anti-CD34 PE (Clone RAM34), anti-CD45 APC (Clone 30-F11), anti-CD71 FITC (Clone C2), anti-CD29 FITC (Clone Ha2/5), anti-CD90 PerCP (Clone OX-7) antibodies, all from BD Biosciences (San Diego, CA, USA). The cells were washed, fixed in 2% formaldehyde in 0.15 M PBS and stored in the refrigerator prior to analysis on the flow cytometer within 24 h. Analyses were performed with a FACSCalibur flow cytometer (BD Biosciences) where 30,000 events were acquired per sample. Data were analyzed using FlowJo v10.1 software (FlowJo, Oregon, USA).

### Multilineage ASC potential

In order to confirm the capacity of ASC to differentiate into mesoderm cell types, we cultured cells with osteogenic, adipogenic, and chondrogenic induction medium, as described below:

Osteogenic differentiation*—*To induce osteogenic differentiation, 1×10^5^ ASC per well were cultured in 6-well plates (Techno Plastic Products AG, Trasadingen, Switzerland) at 37°C in 5% CO_2_ for 14 and 21 days in basal medium supplemented with 0.02 M β-glycerophosphate (Sigma), 5.67 M ascorbic acid (Merck, Darmstadt, Germany) and 10 nM dexamethasone (Sigma). Mineralized nodules in the extracellular matrix assessed by Von Kossa staining assay confirmed osteogenic differentiation. Briefly, cells were fixed in 70% ethanol, incubated with 5% silver nitrate (Vetec, Rio de Janeiro, Brazil) and exposed to ultraviolet light for 1 h. Subsequently, the cells were rinsed with distilled water, 5% sodium thiosulfate (Cinética Química Ltda, Brazil), counterstained with eosin for 40 seconds, and washed with distilled water. The cells were analyzed using the Axio Observer A1 microscope (Zeiss, Göttingen, Germany) at 100X and 400X magnification, and images were captured using the AxioCam MRc camera (Zeiss).

Adipogenic differentiation. To induce adipogenic differentiation, 2×10^5^ ASC were seeded into 6-well plate (Techno Plastic Products AG) and cultured at 37°C in 5% CO_2_ for 14 and 21 days in basal medium supplemented with 0.5 mM isobutylmethyl-xanthine (Sigma), 200 μM indomethacin (Sigma), 1 μM dexamethasone (Aché, São Paulo, Brazil), and 10 μM insulin (Eli Lilly and Company, Indiana, USA). The adipogenic differentiation was analyzed by Oil Red O staining (Thermo Fisher Scientific, Waltham, Massachusetts, USA), an indicator of intracellular lipid accumulation, following the manufacturer’s instructions. Briefly, cells were washed with PBS and fixed in 10% formalin for 1 h, then washed with 60% isopropanol and stained with Oil-Red O (Thermo Scientific) solution in 60% isopropanol for 5 min, rinsed with deionized water, and counterstained with hematoxylin for 1 min. The cells were analyzed using the Axio Observer A1 microscope (Zeiss, Göttingen, Germany) at 100X and 400x magnification, and images were captured using the AxioCam MRc camera (Zeiss, Göttingen, Germany).

Chondrogenic differentiation. To induce chondrogenic differentiation, 5×10^5^ ASC were cultured in a polypropylene conical tube (Falcon) at 37°C in 5% CO_2_ for 14 and 21 days in chondrogenic medium (Stem Pro Chondrogenesis Differentiation–Life Technologies) supplemented with BSF 10%. At the end of each culture time point, a cell pellet was formed and collected for staining of proteoglycans and glycosaminoglycans. Briefly, the pellet was fixed in 10% buffered formaldehyde, embedded in paraffin using a previously described routine technique [49]. Sections of 4 μm were stained with Alcian Blue 8GX 1% in acetic acid, pH 2.5 (Sigma) for 30 min and counterstained with hematoxylin for 1 min. The preparation was analyzed using the Axio Observer A1 microscope (Zeiss, Göttingen, Germany) at 100X and 400X magnification, and images were captured using the AxioCam MRc camera (Zeiss, Göttingen, Germany).

### Study groups

Six to eight-week-old male C57BL/6 mice were infected with *S*. *mansoni* cercariae obtained from *Biomphalaria glabrata* snails, previously infected with miracidia of the L.E. strain, from Belo Horizonte, Brazil. All mice were exposed to 45±5 cercariae on the back skin [50]. After 45 days, they were divided into 2 groups of mice (8 per group) as follows: a) treated orally with PZQ (400mg/Kg) [51], and injected intravenously with PBS 30 days after PZQ treatment (PZQ group); b) treated intravenously with ASC 30 days after PZQ treatment (PZQ/ASC group). A noninfected group (n = 8) was followed at all treatment timelines. Fifteen days after the last treatment (intravenous PBS or ASC), the mice were euthanized by cervical dislocation, and blood samples were collected along with spleens and livers for analysis (Fig 1A). All collected organs were weighed. Adult worms were recovered after infection using a perfusion technique as previously described [52].

### Biodistribution of tdTomato-expressing ASC

To confirm the presence of ASC in the liver, we tracked cells using tdTomato-expressing ASC. C57BL/6 mice were infected with cercaria and treated orally with PZQ as described above. Then, mice were injected intravenously with 3x10^5^ tdTomato-expressing ASC derived from ROSA^mT/mG^ mice. At 1 day, 7 days, and 15 days post ASC injection, the animals were euthanized, and their liver and lungs collected. These organs were fixed in 4% paraformaldehyde overnight at 2°C followed by immersion in 30% sucrose solution for 48 hours at 2°C. The tissues were then embedded in Optimal Cutting Temperature compound (OCT) Tissue Tek (Sakura, CA, USA) and frozen at -80°C to prepare cryosections 20 μm thick using cryostat model CM1850 (Leica, Wetziar, Germany). Tissue sections were counterstained with DAPI (Invitrogen, Carlsbad, California, USA) and the samples were analyzed using the Ti-E C2 plus confocal microscope (Nikon, Minato, Tokyo, Japan) at a magnification of 100x. The images were captured and analyzed using NIS-Elements (Nikon).

### Histological analysis

Livers were excised from sacrificed mice, instantly fixed in 10% formalin in PBS and embedded in paraffin. Then, tissue sections (4 μm) were cut and stained either with hematoxylin and eosin (H&E) or with Masson trichrome to examine the phase and cellular composition of the granulomas, or the extent of hepatic fibrosis, respectively. The phases and cellular composition of granulomas were determined by the analysis of granulomas in five microscopic fields at 100X and 400X magnification using the criteria described in Fig 3A. In the tissue sections stained with Masson´s trichrome, the percentage of the area consisting of collagen of ten non-coalescent granulomas was measured according to a previously described protocol [53]. To assess the number of granulomas, five tissue sections from each lobe of the liver that had been stained with HE were analyzed at 100x magnification, totalizing twenty slides per animal. To evaluate the granuloma volume density, one tissue section from each lobe of the liver stained with HE was analyzed, totalizing four slides per animal. The diameter of all individual granulomas with a single well-defined egg was measured using a 10 objective lens and an ocular scale from the Eclipse e600 microscope (Nikon, New York, EUA). Assuming the spherical shape of granuloma the volume was calculated using the formula to sphere volume (V = R^3^ x Pi x 4/3; R = radius, Pi = 3,14) [54]. The remaining histological analysis was performed using the Axio Observer A1 microscope (Zeiss, Göttingen, Germany). All images were captured using the AxioCam MRc camera (Zeiss, Göttingen, Germany).

### Alanine aminotransferase (ALT) serum levels

ALT was measured by using the transaminase TGP K035 kit (Bioclin, Minas Gerais, Brazil) in accordance with the manufacturer’s instructions. Absorbance was determined at 505 nm using a Spectramax M5 (Molecular Devices, California, USA).

### Phenotypic characterization of T lymphocytes

The spleen and the liver were collected and processed separately. The right medial lobe of the liver was minced and incubated with 0.15% type II collagenase (Life Technologies) for 1 hour at 37°C. Adding RPMI 1640 media stopped the enzymatic action. The cells were collected at 350 × *g* for 5 min at 4°C, suspended in 30 mL of RPMI 1640 media, and pellet at 60 × *g* for 3 min at 4°C. The supernatant containing the leukocytes was collected. The spleen samples were macerated in RPMI 1640 media (Gibco) containing 40 μg/mL of gentamicin. The cell suspensions obtained from liver or spleen were passed once through a 70 μm-cell strainer (BD Biosciences) and transferred to a sterile 50 mL tube. Contaminating erythrocytes were removed by hypotonic lysis in distilled water, and isotonicity was restored by adding 1.5 M PBS. Cells were pelleted at 350 × *g* for 10 min and then suspended in 1 mL of RPMI 1640 medium containing 40 μg/mL of gentamicin. Next, the cells were stained in Turk's solution (1:20) and counted in using a Neubauer chamber. 1 × 10^6^ viable cells were added in each well of U-bottom 96-well plates. The cells were suspended in 1% bovine serum albumin (Sigma) in 0.15 M PBS solution. For flow cytometry analysis, the cultured cells were stained for 30 min at 4°C with anti-CD3 FITC (Clone 145-2C11) (BD Pharmigen), anti-CD4 PerCP (Clone RM4-5) (BD Pharmigen), anti-CD8 PerCP (Clone 53–6.7) (BD Pharmigen), anti-CD69 PE (Clone H1.2F3) (BD Pharmigen), anti-CD25 PE (Clone PC61) (BD Pharmigen), anti-CD28 APC (Clone 37.51) (eBiosciense) and anti-CTLA4 APC (Clone UC10-4B9) (eBiosciense) antibodies. After staining, the cells were fixed with 2% formaldehyde in 0.15 M PBS and stored in the refrigerator for flow cytometry analysis within 24h. Analyses were performed with a FACSCalibur flow cytometer (BD Biosciences) and 30,000 events were acquired per sample. The data were analyzed using FlowJo v10.1 software (FlowJo, Oregon, USA).

### Cytokine detection

Cytokine levels were analyzed in mouse serum and in the collected fragment of the liver. For *in situ* measuring, a lobe of the liver was weighed on an analytical balance and transferred to a 1.5 mL tube (Eppendorf, Hamburg, Germany) containing 500 μL of complete EDTA-free protease inhibitor (Roche, Basel, Switzerland). The lobes were immediately macerated using an automatic Pellet Pestle Motor macerator (Thomas cientific, NJ, USA) and stored at -80°C for further analysis by flow cytometry. The serum samples were obtained from blood collected from the brachial plexus and centrifuged 350 x *g* for 5 minutes. All serum samples were stored at -80°C until tested individually for cytokines. The levels of IL-10, IL-17α, TNF-α, IFN-γ, IL-6, IL-4 and IL-2 were quantified in both samples (serum and *in situ*) using the BD Cytometric Bead Array (CBA) Mouse Th1/Th2/Th17 Cytokine Kit (BD Bioscience, San Diego, CA, USA) following the manufacturer's instructions. Liver cytokine data were acquired using the FACSverse flow cytometer (BD), analyzed using CellQuest (BD) software, and the results expressed in pg/100mg. The serum sample data were acquired using the FASCS Calibur flow cytometer (BD), analyzed using BD Cytometric Bead Array Analysis CBA (BD), and the results expressed in pg/mL. We also analyzed the frequency of mice producing high levels of pro- and anti-inflammatory cytokines in each group (PZQ and PZQ/ASC). Briefly, we first calculated the global median value of whole universe of data (NI + PZQ + PZQ/ASC) for each cytokine (S2 Fig). Then, the global median value was used as the cut-off to classify mice as a “low” (cytokine level under the cut-off) or “high” (cytokine level up the cut-off) producer of a given cytokine [55]. The number of mice with cytokine levels above the cut-off value was reported in radar plots as frequency of high producer mice for each group (PZQ and PZQ/ASC).

### Statistical analyses

Bartlett's test was used to evaluate equal variances and Kolmogorov-Smirnov test—to evaluate normality of data distribution. The Mann-Whitney test was used to compare related samples. Differences were considered statistically significant when a *p* value ≤ 0.05 was obtained. Prism 8 software package (Graphpad Software, California, USA) was used for statistical tests.

## Results

### ASC characterization

The cultured cells grew homogeneously, showing a monolayer consisting of adherent cells, forming colonies with fibroblast-like morphology (S1A Fig) with all traits of MSC. Simultaneously, the expression of hematopoietic and mesenchymal cell markers was investigated and showed phenotypic characteristic that match with those previously described for MSC. CD34 and CD45 were expressed in 2.12% and 1.81% of cells, respectively. In contrast, the percentage of cells expressing CD71, CD29, and CD90, all mesenchymal cell markers, was higher than 42% (S1C Fig). ASC were further analyzed for their capacity to differentiate into osteoblastic, adipoblastic and chondroblastic lineages. Osteogenic differentiation was confirmed by mineralized nodules in the extracellular matrix, evidenced by Von Kossa staining (S1D Fig). ASC cultured with adipogenic inductors showed intracellular lipid vacuoles evidenced by Oil Red O staining (S1D Fig). Chondrogenic differentiation was confirmed by glycosaminoglycans in the extracellular matrix, evidenced by Alcian blue staining (S1D Fig). Our results have confirmed that the cells isolated from inguinal adipose tissue from C57BL/6 mice were MSCs as expected.

### Tracking of ASC

In order to investigate the presence of ASC in the liver, we used tdTomato expressing-ASC extracted from mouse ROSA^mT/mG^. We analyzed lung and liver tissues after 1, 7, and 15 days following ASC^tdTomato+^ intravenous injection. The tdTomato^+^ ASC were detected first in the lung, and later in the liver, 15 days after transfer (Fig 1B).

### ASC therapy reduced the volume of granuloma

**Fig 2:**
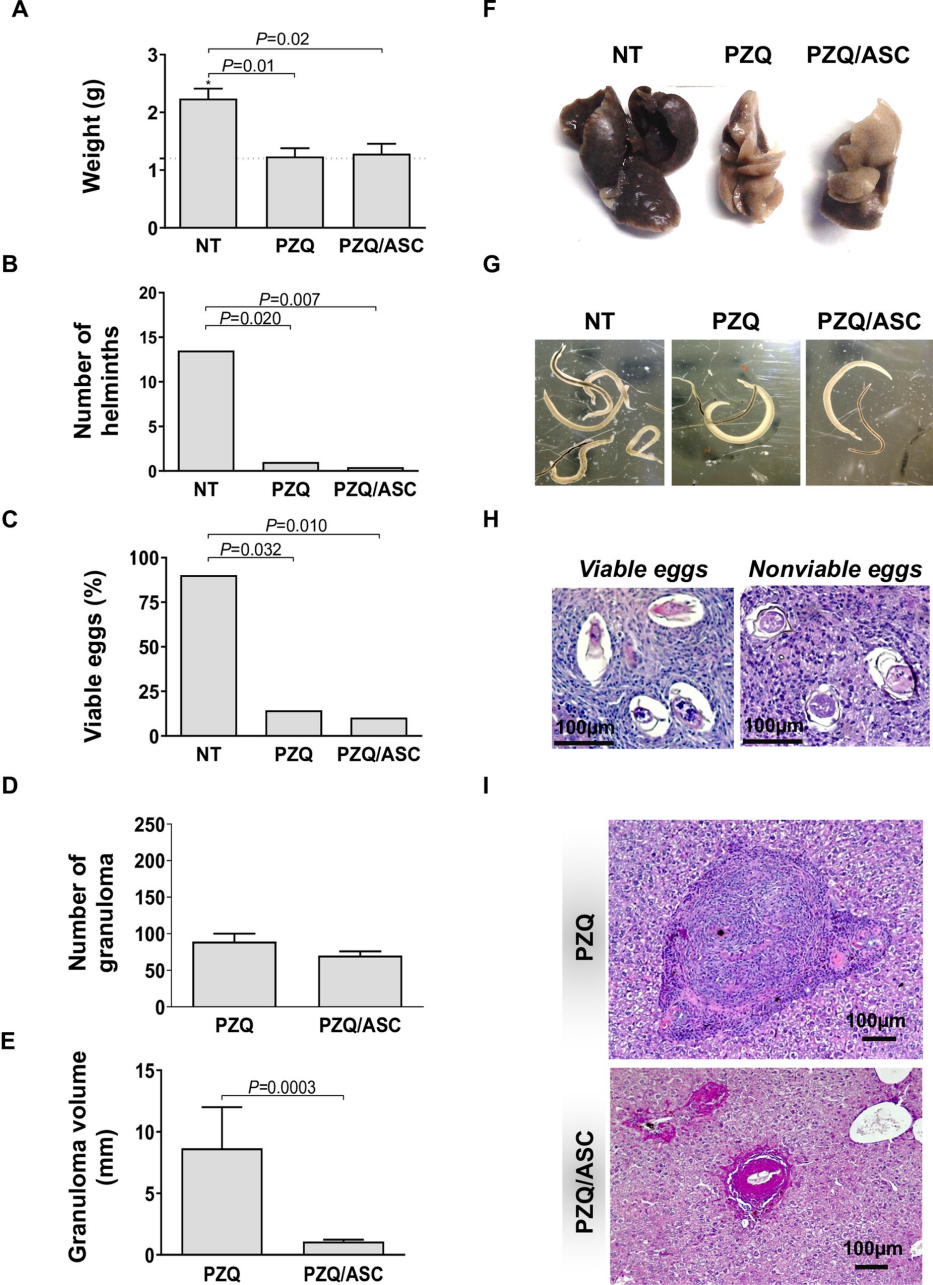
ASC effect in *S*. *mansoni* infection. Panels A-E (mean ± standard deviation) show the liver weight (A), number of helminths recovered from mesenteric vessels (B), percentage of viable eggs (C), number (D) and volume (E) of granulomas in the liver from *Schistosoma*-infected C57BL/6 mice treated with PZQ coadministered with ASC. Dashed lines represent the mean of non-infected mice. The horizontal lines indicate differences statistically significant (p<0.05) between groups. Representative images of the liver (F), helminths recovered (G), viable/nonviable eggs (H) and granulomas (I) stained with HE. NT = infected not treated group; PZQ = infected treated with praziquantel group; PZQ/ASC = infected treated with praziquantel plus adipose tissue-derived stem cells group.

We have analyzed the effect of PZQ and ASC treatment on *S*. *mansoni* infections. Our results show that the group treated with PZQ/ASC presented liver weights (Fig 2A and 2F), numbers of parasites (Fig 2B and 2G), and percentage of viable eggs (Fig 2C and 2H) similar to group treated with PZQ alone. Interestingly, the volume of granuloma (Fig 2E and 2I) was significantly lower (*P* = 0.0003) in mice treated with PZQ/ASC compared to the PZQ group.

### ASC therapy accelerated the progression of hepatic granulomas in PZQ-treated mice

**Fig 3:**
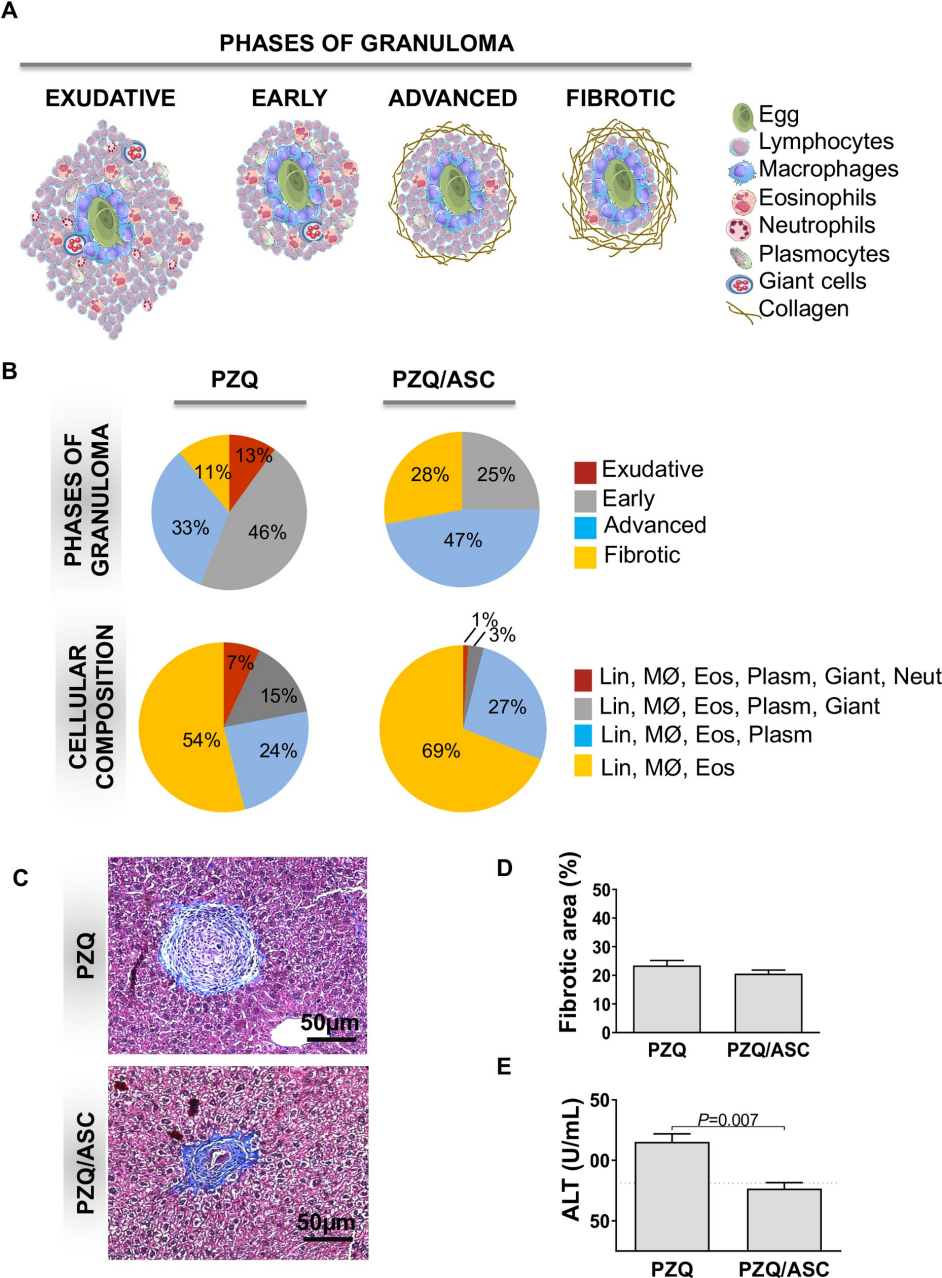
ASC therapy effect on granulomas and ALT levels. Panel A shows a schematic representation of the phases and cellular composition of granulomas. The exudative phase is characterized by the establishment and organization of the granulomatous reaction with focal destruction of the vessel walls involved and/or adjacent parenchymal and abundant presence of eosinophils, macrophages and other immunologic cells. The early phase is characterized by the presence of giant cells and the dissociation of collagen fibers. An increase in collagen characterizes the advanced phase. Fibrosis is characterized by a significant decrease in cellular composition and thickening of the collagen layer. Panel B shows the comparative analysis of the phase and cellular composition of the granulomas in PZQ and PZQ/ASC groups. Panel C shows representative images from the liver granulomas stained with Masson’s trichrome. Panel D shows fibrotic area in the liver. Panel E shows the levels of ALT in sera of *Schistosoma*-infected C57BL/6 mice treated with PZQ or PZQ/ASC, expressed as the mean ± standard deviation. The horizontal lines indicate statistically significant differences between the groups (p<0.05). PZQ = Praziquantel-treated group. PZQ/ASC = Praziquantel plus adipose tissue-derived stem cells-treated group.

Schistosomiasis is characterized by granulomatous reactions that cause significant pathology of several organs, mainly in the liver. We performed a comparative analysis of the phase and cellular constitution of granulomas in histological sections of the liver between all groups (Fig 3A and 3B). Most granulomas from the PZQ group were in the early phase, rich in lymphocytes, macrophages, eosinophils, plasmocytes and giant cells (Fig 3B). On the other hand, the animals that were treated with the combination of PZQ/ASC presented mainly granulomas in the advanced phase, rich in lymphocytes, macrophages, eosinophils and plasmocytes (Fig 3B). The PZQ/ASC group also showed a large percentage of granulomas in the fibrotic phase, rich in lymphocytes, macrophages and eosinophils (Fig 3B and 3D). Masson’s trichrome staining estimated that similar collagen deposition in hepatic granulomas in PZQ and PZQ/ASC groups (Fig 3C and 3D). These findings suggest that ASC therapy alongside PZQ treatment induces a faster resolution of hepatic granulomas in *S*. *mansoni*-infected mice.

### ALT levels were reduced in the group treated with PZQ and ASC

To monitor the extent of liver damage after *S*. *mansoni* infection, we measured the levels of ALT in sera of animals from the different groups. The results show that the levels of ALT were significantly lower in the PZQ/ASC group compared to the PZQ only group (Fig 3E), suggesting that ASC injection after PZQ treatment reduces liver damage caused by *S*. *mansoni* infection.

### CD4+ and CD8+ T lymphocytes activation profile after ASC therapy

**Fig 4:**
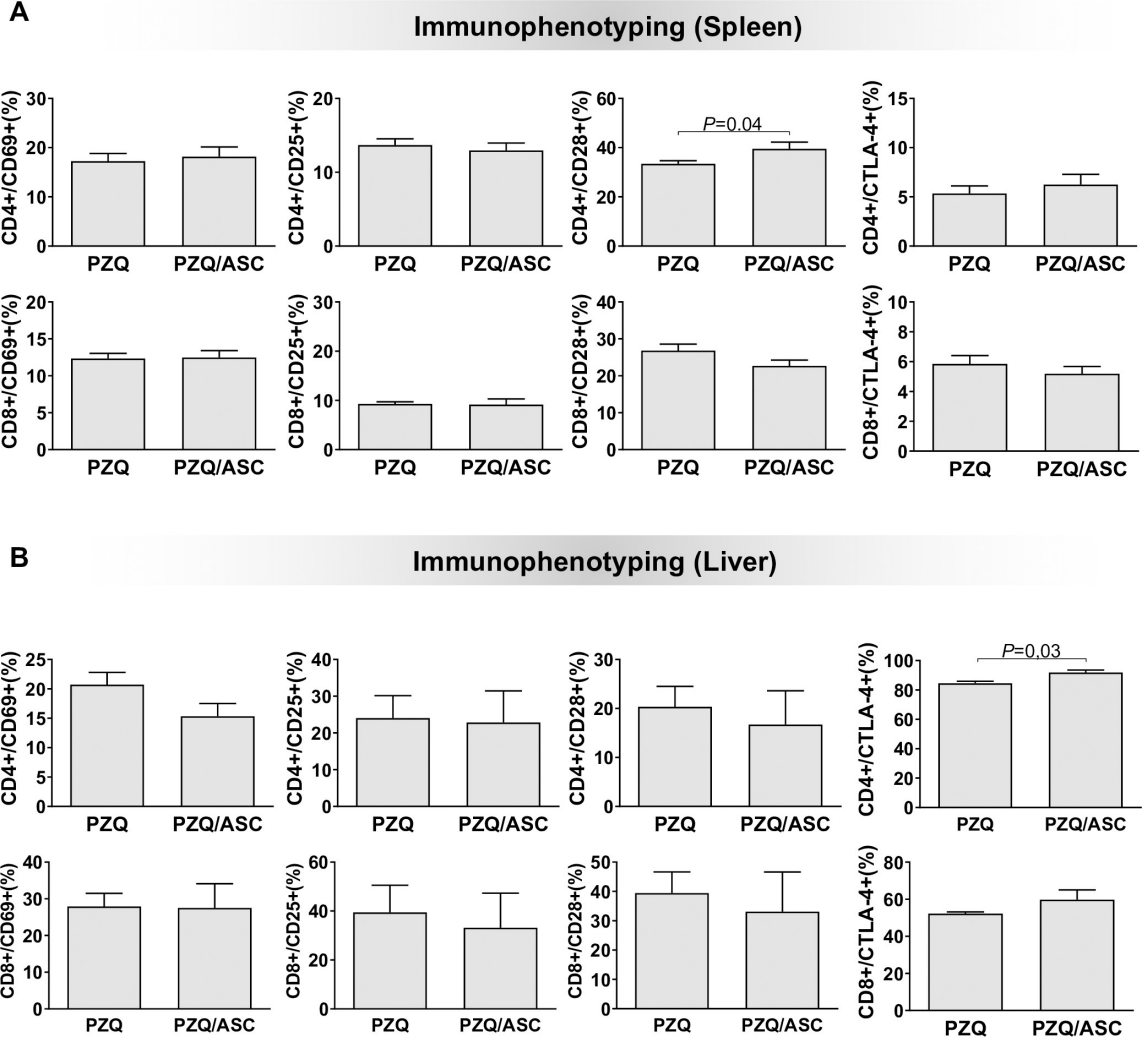
T cell activation before and after ASC therapy. Frequency of CD4^+^ and CD8^+^ T lymphocytes expressing CD69, CD25, CD28 and CTLA-4 in spleen (A) and liver (B) cells. The data are shown as means ± standard deviation. Horizontal lines indicate statistically significant differences (p<0.05). PZQ = Praziquantel-treated group; PZQ/ASC = Praziquantel plus adipose tissue-derived stem cells-treated group.

Under some conditions, MSC can modulate the immune response. To test whether these cells can induce the regulation of the immune response during *S*. *mansoni* infection, we have analyzed the percentage of CD4^+^ and CD8^+^ T lymphocytes expressing CD25, CD69, CD28 and CTLA-4 in the spleen and liver of *S*. *mansoni*-infected C57BL/6 mice after ASC treatment. We observed significant expression of the CD28 and CTLA-4 on CD4+ lymphocytes from the spleen (Fig 4A) and the liver (Fig 4B), respectively. CD8+ T cells showed similar stimulation before and after ASC treatment in both organs (Fig 4). Together, our results show that ASC treatment alters the activation profile of CD4^+^ T cells in the spleen and the liver from *S*. *mansoni*-infected mice treated with PZQ.

### Cytokine profile after ASC therapy

**Fig 5:**
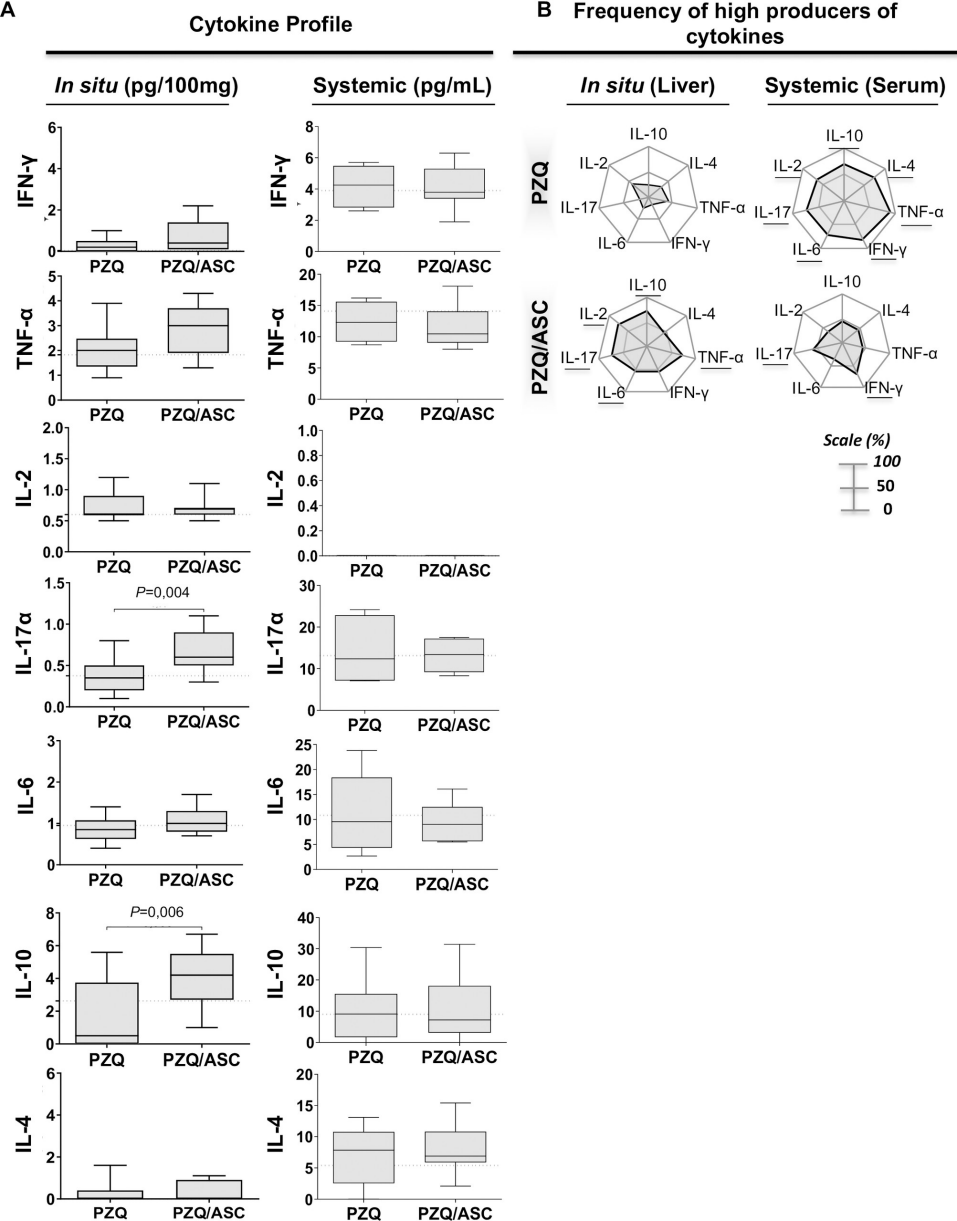
Cytokine profile before and after ASC therapy. Panel A shows cytokine levels *in situ* (liver) and systemic (in serum) from *Schistosoma*-infected C57BL/6 mice with PZQ only or PZQ/ASC treatment. Panel B shows radar charts summarizing the percentage of high cytokine producers of each group in comparison to the global median value of whole universe of data (NI + PZQ + PZQ/ASC) for each cytokine (S2 Fig). When the frequency of high producers was greater than 50% (on a scale of 0–100%), the result was highlighted. The data are shown as means ± standard deviation. The horizontal lines indicate statistically significant differences (p<0.05). PZQ = Praziquantel-treated group; PZQ/ASC = Praziquantel plus adipose tissue-derived stem cells-treated group.

The levels of IL-2, IL-10, TNF-α, INF-γ, IL-6, IL-17A and IL-4 were measured in serum (systemic) and livers (*in situ*) of mice treated with PZQ or PZQ/ASC. Serum cytokine levels were not significantly different between the treatment groups (Fig 5A). Liver cytokine profiles showed high levels of IL-17A and IL-10 induced by ASC treatment (Fig 5A). When we analyzed the frequency of high cytokine producers, we observed that the PZQ/ASC treatment induced high frequency (>50%) of pro-inflammatory cytokines in the liver and PZQ treatment alone induced high frequency (>50%) of cytokines in serum (Fig 5B).

## Discussion

In our study, we have hypothesized that during infection with *S*. *mans*oni, treatment with PZQ followed by ASC therapy leads to a faster resolution of hepatic inflammation and fibrosis in experimental murine models of the disease. First, we have confirmed the multipotential of ASC as MSC through phenotypic and functional characterization assays, following the criteria standardized by the International Society of Cell Therapy [56]. The ASC, in our study, showed a fibroblast-like morphology as well as expression of cell surface markers consistent with MSC [57]. Functional characterization tests showed the multipotential of ASC to differentiate into osteogenic, adipogenic and chondrogenic cell lines, also characteristic of MSC [23].

To demonstrate that ASC preferentially migrated to the injury sites following intravenous administration [58], we injected tdTomato^+^ ASC intravenously and screened for red fluorescent cells in the lungs and the liver using a confocal microscope. Within 1 and 7 days of ASC injection, red fluorescent cells were found in the lungs, but not in the liver. Within 15 days of ASC injection, red fluorescent cells could be seen in the liver, but not in the lungs. These results suggest that tdTomato^+^ ASC were first retained in the lungs due to the blood of the venous circulation and later migrated to the liver, possible due to the damage caused by *S*. *mansoni* eggs. Studies evaluating the ability of stem cells to migrate to the injured liver have conflicting findings. Some of them reported that the MSC gradually accumulated in the liver [59, 60], while in another study showed that transplanted MSC reach a maximal amount in the liver 24 hours post-infusion and gradually decrease thereafter [61]. Because we have observed ASC tdTomato^+^ in the liver on the 15th day after the injection, we surmize that their effect might be local, through cell-to-cell contact and/or secretion of soluble factors.

Many studies reported an anti-inflammatory activity of MSC modulated by high levels of TNF-α, IFN-γ, and IL-1 cytokines. In this study, we investigated the potential for ASC to control inflammation and liver fibrosis due to the *S*. *mansoni* infection following conventional treatment with PZQ. To evaluate the putative effect of ASC on the control of inflammation and induction of tissue regeneration, we designed our study linking the known anti-parasite effect of PZQ with the ASC properties to determine whether combined PZQ and ASC therapy would induce a faster recovery than the treatment with PZQ alone. The modulation of granulomas and tissue repair in some *S*. *mansoni* infected individuals [62] can take a long time when PZQ is used as the only therapy [19, 63]. Therefore, the possible effect of ASC on controlling inflammation and reducing liver fibrosis could be an important adjunct treatment.

As expected, PZQ treatment showed efficacy in the decrease or elimination of the number of adult worms, and consequently decrease in the number of new eggs in the mesenteric circulation [64]. As shown in other studies, the majority of the *S*. *mansoni* eggs recovered from treated animals were not viable [65]. The large number of non-viable eggs after PZQ treatment may have contributed to the reduction of granulomatous inflammation [7, 66], causing a decrease in the liver weight in the PZQ-treated group. These same effects were observed in the group of animals treated with PZQ in combination with ASC, i.e. improvement in liver weight, number of helminths, and percentage of non-viable eggs, showing that ASC therapy did not exceed the effect of PZQ treatment alone.

To investigate whether ASC treatment altered the microscopic structure of the liver, we analyzed tissue sections stained with hematoxylin & eosin. Our data showed that animals treated with PZQ in combination with ASC had a similar number of granulomas compared to mice treated only with PZQ. However, the granulomas from animals treated with PZQ combined with ASC were reduced in volume compared to mice treated with PZQ only. Although previous studies have shown that bone-marrow-derived mesenchymal cells (BM-MSC) can lead to the reduction of hepatic granuloma diameter in *S*. *mansoni*-infected BALB/c mice [46] as well as in the *S*. *japonicum* murine model, treated with PZQ [43], our study is the first to show that adipose tissue-derived stem cell therapy reduces the volume of hepatic granulomas resulting from *S*. *mansoni* infection.

To more accurately characterize the granulomas of animals subjected to the different treatment regimens and the possible causes of this reduced volume, we analyzed the phase and cellular composition of the hepatic granulomas. The development of *Schistosoma* granulomas has been shown to follow sequential phases: a) exudative; b) early; c) advanced; and d) fibrotic. Throughout these phases, the complexity of cells decreases and leads to a curative fibrotic phase where a large number of inflammatory cells are gradually replaced by collagen [53], which is the main cause of the severe pathology. We observed that animals treated with PZQ presented a large number of early and advanced granulomas that were rich in lymphocytes, macrophages, eosinophils, plasmocytes and giant cells while mice treated with PZQ and ASC presented more advanced and fibrotic granulomas that were rich in lymphocytes, macrophages, eosinophils, and plasmocytes. The granulomatous reaction that is observed in animals infected with *S*. *mansoni*, is due to the immune reaction to secreted egg antigens, which are known to be highly cytotoxic to hepatic cells. After deposition of the eggs in the tissues, they are rapidly surrounded by infiltrates of inflammatory cells, resulting in granulomatous reaction and fibrosis [67]. This is a dynamic process in which the diameter and cellular composition of the lesions change over time and are coordinated by the influence of a network of inflammatory mediators [67]. Our results showed that administration of ASC after PZQ treatment promoted an accelerated process to the fibrotic phase. We further investigated whether this effect changed the outcome of the granuloma lesions by analysis of the percentage of the fibrotic area in the granulomas from PZQ- and PZQ/ASC-treated groups. We could only evaluate the fibrosis using one semi-quantitative method and did not observe significant alterations in collagen deposition after ASC treatment. Previous studies using treatment with BM-MSC showed a reduced percentage of the fibrotic area in the experimental hepatic schistosomiasis model [42, 44, 46]. Fibrosis induced by *S*. *japonicum* was also ameliorated by BM-MSC plus PZQ therapy [43]. It has been shown that there are differences in the functional properties and the differentiation capacity between distinct populations of stem cells. The signature of BM-MSC indicates that they are primed toward the developmental processes of tissues and organs derived from the mesoderm and endoderm, while ASC appear to be highly enriched in immune-related genes [68, 69]. The BM-MSC and ASC antifibrotic therapy are similarly effective at attenuating carbon tetrachloride^-^induced liver fibrosis in animal models by inhibiting the activation and proliferation of hepatic stellate cells (HSCs), as well as promoting the apoptosis of HSCs [70].

To investigate the effect of PZQ/ASC treatment on liver function, we evaluated the levels of circulating ALT in the animals of the different groups. ALT is a cytosolic enzyme that is found in high concentrations in the liver and is released into the extracellular environment when hepatocellular lesions occur [71]. Our results showed that mice treated with ASC and PZQ expressed lower serum levels of ALT than mice treated with PZQ only. Furthermore, the ALT levels from the PZQ/ASC group were similar to uninfected animals, indicating that ASC injection was able to reduce hepatocyte damage. Similar results were also obtained after treatment with BM-MSC in the murine model of schistosomiasis [44, 46]. It is possible that ASC treatment reduced the injury to hepatocytes leading to the decreased serum levels of ALT observed in PZQ/ASC-treated group.

The histopathological data indicated that ASC treatment induced a fast resolution of granulomas in the liver. To investigate whether this result impacted the immune response or not, we analyzed the frequency of CD4^+^ and CD8^+^ T lymphocytes expressing CD69, CD25, CD28 and CTLA-4, *in situ* (spleen and liver), and the systemic (serum) and *in situ* (liver) levels of IFN-γ, TNF-α, IL-2, IL-6, IL-17A, IL-4 and IL-10. ASC treatment interfered with the immune response mainly in the liver where we found a high frequency of CD4^+^ T cells expressing CTLA-4 as well as high levels of IL-10 and IL-17 cytokines. Although Th17 cells are associated with aggravation of the pathology in murine model of *S*. *mansoni* infection [72], the high levels of IL-17A and IL-10 may reflect in the regulation of Th2 response, reducing the granuloma size and inflammation, and more efficient granuloma resolution. Furthermore, IL-10 can contribute to the anti-inflammatory environment, and has been correlated with reduction of granulomas in patients with *S*. *mansoni* [6, 17].

In conclusion, our results demonstrate that *S*. *mansoni*-infected mice treated with PZQ/ASC had smaller granulomas, less tissue damage and faster evolution to the curative fibrotic phase, suggesting that ASC therapy has immunomodulatory effect. Although the findings are promising, further studies using the chronic model of schistosomiasis are required to determine the potential therapeutic application of ASC in schistosomiasis. These findings are encouraging and supportive for the call for innovative therapeutic approaches using MSC to minimize hepatic lesion caused by *S*. *mansoni* infection.

## Supporting information

S1 Fig Phenotypic and functional characterization of adipose tissue-derived stem cells (ASC). (A) Fibroblast-like morphology of ASC at passage 3 on culture; scale bar 100μm. (B) Viability of ASC evaluated by 3-(4,5-dimethylthiazol-2-yl)-2,5-diphenyltetrazolium bromide (MTT) assay at an optical density (O.D.) at 595nm. Data are presented as mean ± standard error of the mean. (C) Histograms for markers expressed (CD71, CD29, and CD90) or not (CD34 and CD45) by ASC. (D) Osteogenic, adipogenic and chondrogenic multilineage potential of ASC after 14 and 21 days on culture with respective inductors medium.
(TIF)

S2 Fig Cut-off thresholds used to segregate mice according to low or high levels of cytokines. The global median value of all data (NI + PZQ + PZQ/ASC) for each cytokine was calculated and used as the cut-off to classify mice as a “low” (cytokine level under the cut-off) or “high” (cytokine level above the cut-off) producer of a given cytokine.
(TIF)

S3 Fig Graphical abstract. Differences between conventional treatment for schistosomiasis mansoni using PZQ and the proposed treatment of a combination therapy PZQ/ASC. There was a significant decrease in the size of the granulomas in the liver of mice receiving the combination treatment compared to the animals that received only PZQ. In addition, a decrease in serum ALT levels was observed, indicating a reduction of tissue damage. In addition, our results showed that 15 days after injection, ASC were found in the liver. Nonetheless, the mechanics used by ASCs to perform such functions still remain unclear. The complexity of the formation and progression of the granulomatous reaction is one of the reasons why a robust anti-inflammatory therapy has not yet been developed. It may be that a single anti-inflammatory "magic bullet" is simply unable to overcome such complex diseases. Therefore, the presumed effects of ASCs together such as immunomodulation, hepatoprotection, stimulation of hepatic cell proliferation and even differentiation of ASC in hepatocytes may be the answer to the phenomena observed in our study.
(TIF)

## References

[1] Zoni AC, Catala L, Ault SK. Schistosomiasis Prevalence and Intensity of Infection in Latin America and the Caribbean Countries, 1942–2014: A Systematic Review in the Context of a Regional Elimination Goal. PLoS Negl Trop Dis. 2016;10(3):e0004493 Epub 2016/03/24. 10.1371/journal.pntd.0004493 27007193 PMC4805296

[2] Schistosomiasis. WHO. 2017. doi: /entity/mediacentre/factsheets/fs115/en/index.html.

[3] Vieira P, Miranda HP, Cerqueira M, Delgado Mde L, Coelho H, Antunes D, et al Latent schistosomiasis in Portuguese soldiers. Mil Med. 2007;172(2):144–6. Epub 2007/03/16. 10.7205/milmed.172.2.144 . 17357767

[4] Mathew RC, Boros DL. Anti-L3T4 antibody treatment suppresses hepatic granuloma formation and abrogates antigen-induced interleukin-2 production in *Schistosoma mansoni* infection. Infect Immun. 1986;54(3):820–6. Epub 1986/12/01. 10.1128/IAI.54.3.820-826.1986 3096893 PMC260243

[5] Iacomini J, Ricklan DE, Stadecker MJ. T cells expressing the gamma delta T cell receptor are not required for egg granuloma formation in schistosomiasis. Eur J Immunol. 1995;25(4):884–8. Epub 1995/04/01. 10.1002/eji.1830250404 . 7737289

[6] Silveira AM, Gazzinelli G, Alves-Oliveira LF, Bethony J, Gazzinelli A, Carvalho-Queiroz C, et al Human schistosomiasis mansoni: intensity of infection differentially affects the production of interleukin-10, interferon-gamma and interleukin-13 by soluble egg antigen or adult worm antigen stimulated cultures. Trans R Soc Trop Med Hyg. 2004;98(9):514–9. Epub 2004/07/15. 10.1016/j.trstmh.2003.11.009 . 15251399

[7] Hams E, Aviello G, Fallon PG. The *Schistosoma* granuloma: friend or foe? Front Immunol. 2013;4:89 Epub 2013/04/19. 10.3389/fimmu.2013.00089 23596444 PMC3625856

[8] Hatz CF, Vennervald BJ, Nkulila T, Vounatsou P, Kombe Y, Mayombana C, et al Evolution of *Schistosoma haematobium*-related pathology over 24 months after treatment with praziquantel among school children in southeastern Tanzania. Am J Trop Med Hyg. 1998;59(5):775–81. Epub 1998/12/05. 10.4269/ajtmh.1998.59.775 . 9840596

[9] Parra JC, Doughty B, Colley DG, Gazzinelli G. Human schistosomiasis mansoni: studies on *in vitro* granuloma modulation. Mem Inst Oswaldo Cruz. 1992;87 Suppl 5:79–81. Epub 1992/01/01. 10.1590/s0074-02761992000900011 . 1342721

[10] Pearce EJ, MacDonald AS. The immunobiology of schistosomiasis. Nat Rev Immunol. 2002;2(7):499–511. Epub 2002/07/03. 10.1038/nri843 . 12094224

[11] Everts B, Perona-Wright G, Smits HH, Hokke CH, van der Ham AJ, Fitzsimmons CM, et al Omega-1, a glycoprotein secreted by *Schistosoma mansoni* eggs, drives Th2 responses. J Exp Med. 2009;206(8):1673–80. Epub 2009/07/27. 10.1084/jem.20082460 19635864 PMC2722183

[12] Everts B, Hussaarts L, Driessen NN, Meevissen MH, Schramm G, van der Ham AJ, et al Schistosome-derived omega-1 drives Th2 polarization by suppressing protein synthesis following internalization by the mannose receptor. J Exp Med. 2012;209(10):1753–67, S1. Epub 2012/09/10. 10.1084/jem.20111381 22966004 PMC3457738

[13] Steinfelder S, Andersen JF, Cannons JL, Feng CG, Joshi M, Dwyer D, et al The major component in schistosome eggs responsible for conditioning dendritic cells for Th2 polarization is a T2 ribonuclease (omega-1). J Exp Med. 2009;206(8):1681–90. Epub 2009/07/27. 10.1084/jem.20082462 19635859 PMC2722182

[14] Hoffmann KF, Cheever AW, Wynn TA. IL-10 and the dangers of immune polarization: excessive type 1 and type 2 cytokine responses induce distinct forms of lethal immunopathology in murine schistosomiasis. J Immunol. 2000;164(12):6406–16. Epub 2000/06/08. 10.4049/jimmunol.164.12.6406 . 10843696

[15] Falcao PL, Malaquias LC, Martins-Filho OA, Silveira AM, Passos VM, Prata A, et al Human Schistosomiasis mansoni: IL-10 modulates the *in vitro* granuloma formation. Parasite Immunol. 1998;20(10):447–54. Epub 1998/11/03. 10.1046/j.1365-3024.1998.00166.x . 9797505

[16] Alves Oliveira LF, Moreno EC, Gazzinelli G, Martins-Filho OA, Silveira AM, Gazzinelli A, et al Cytokine production associated with periportal fibrosis during chronic schistosomiasis mansoni in humans. Infect Immun. 2006;74(2):1215–21. Epub 2006/01/24. 10.1128/IAI.74.2.1215-1221.2006 16428771 PMC1360316

[17] Teixeira-Carvalho A, Martins-Filho OA, Peruhype-Magalhaes V, Silveira-Lemos D, Malaquias LC, Oliveira LF, et al Cytokines, chemokine receptors, CD4+CD25HIGH+ T-cells and clinical forms of human schistosomiasis. Acta Trop. 2008;108(2–3):139–49. Epub 2008/06/07. 10.1016/j.actatropica.2008.04.010 . 18534548

[18] Crellen T, Walker M, Lamberton PH, Kabatereine NB, Tukahebwa EM, Cotton JA, et al Reduced Efficacy of Praziquantel Against *Schistosoma mansoni* Is Associated With Multiple Rounds of Mass Drug Administration. Clin Infect Dis. 2016;63(9):1151–9. Epub 2016/07/30. 10.1093/cid/ciw506 27470241 PMC5064161

[19] Coutinho A, Domingues AL. Specific treatment of advanced schistosomiasis liver disease in man: favourable results. Mem Inst Oswaldo Cruz. 1987;82 Suppl 4:335–40. Epub 1987/01/01. 10.1590/s0074-02761987000800064 . 3151109

[20] Friedenstein AJ, Chailakhyan RK, Latsinik NV, Panasyuk AF, Keiliss-Borok IV. Stromal cells responsible for transferring the microenvironment of the hemopoietic tissues. Cloning *in vitro* and retransplantation *in vivo*. Transplantation. 1974;17(4):331–40. Epub 1974/04/01. 10.1097/00007890-197404000-00001 . 4150881

[21] Caplan AI. Mesenchymal stem cells. J Orthop Res. 1991;9(5):641–50. 10.1002/jor.1100090504 . 1870029

[22] Uccelli A, Moretta L, Pistoia V. Mesenchymal stem cells in health and disease. Nature Reviews Immunology. 2008;8(9):726–36. 10.1038/nri2395 19172693

[23] Pittenger MF, Mackay AM, Beck SC, Jaiswal RK, Douglas R, Mosca JD, et al Multilineage potential of adult human mesenchymal stem cells. Science. 1999;284(5411):143–7. Epub 1999/04/02. 10.1126/science.284.5411.143 . 10102814

[24] Izadpanah R, Trygg C, Patel B, Kriedt C, Dufour J, Gimble JM, et al Biologic properties of mesenchymal stem cells derived from bone marrow and adipose tissue. J Cell Biochem. 2006;99(5):1285–97. Epub 2006/06/24. 10.1002/jcb.20904 16795045 PMC4048742

[25] Zuk PA, Zhu M, Mizuno H, Huang J, Futrell JW, Katz AJ, et al Multilineage cells from human adipose tissue: implications for cell-based therapies. Tissue Eng. 2001;7(2):211–28. Epub 2001/04/17. 10.1089/107632701300062859 . 11304456

[26] Jones S, Horwood N, Cope A, Dazzi F. The antiproliferative effect of mesenchymal stem cells is a fundamental property shared by all stromal cells. J Immunol. 2007;179(5):2824–31. Epub 2007/08/22. 10.4049/jimmunol.179.5.2824 . 17709496

[27] Di Nicola M, Carlo-Stella C, Magni M, Milanesi M, Longoni PD, Matteucci P, et al Human bone marrow stromal cells suppress T-lymphocyte proliferation induced by cellular or nonspecific mitogenic stimuli. Blood. 2002;99(10):3838–43. Epub 2002/05/03. 10.1182/blood.v99.10.3838 . 11986244

[28] Wei X, Yang X, Han ZP, Qu FF, Shao L, Shi YF. Mesenchymal stem cells: a new trend for cell therapy. Acta Pharmacol Sin. 2013;34(6):747–54. Epub 2013/06/06. 10.1038/aps.2013.50 23736003 PMC4002895

[29] Horwitz EM, Prockop DJ, Fitzpatrick LA, Koo WW, Gordon PL, Neel M, et al Transplantability and therapeutic effects of bone marrow-derived mesenchymal cells in children with osteogenesis imperfecta. Nat Med. 1999;5(3):309–13. Epub 1999/03/23. 10.1038/6529 . 10086387

[30] Le Blanc K, Frassoni F, Ball L, Locatelli F, Roelofs H, Lewis I, et al Mesenchymal stem cells for treatment of steroid-resistant, severe, acute graft-versus-host disease: a phase II study. Lancet. 2008;371(9624):1579–86. Epub 2008/05/13. 10.1016/S0140-6736(08)60690-X . 18468541

[31] Weng JY, Du X, Geng SX, Peng YW, Wang Z, Lu ZS, et al Mesenchymal stem cell as salvage treatment for refractory chronic GVHD. Bone Marrow Transplant. 2010;45(12):1732–40. Epub 2010/09/08. 10.1038/bmt.2010.195 20818445 PMC3035976

[32] Tabbara IA, Zimmerman K, Morgan C, Nahleh Z. Allogeneic hematopoietic stem cell transplantation: complications and results. Arch Intern Med. 2002;162(14):1558–66. Epub 2002/07/19. 10.1001/archinte.162.14.1558 . 12123398

[33] Zhang S, Ge J, Sun A, Xu D, Qian J, Lin J, et al Comparison of various kinds of bone marrow stem cells for the repair of infarcted myocardium: single clonally purified non-hematopoietic mesenchymal stem cells serve as a superior source. J Cell Biochem. 2006;99(4):1132–47. Epub 2006/06/24. 10.1002/jcb.20949 . 16795039

[34] Sun LY, Zhang HY, Feng XB, Hou YY, Lu LW, Fan LM. Abnormality of bone marrow-derived mesenchymal stem cells in patients with systemic lupus erythematosus. Lupus. 2007;16(2):121–8. Epub 2007/04/04. 10.1177/0961203306075793 . 17402368

[35] Thakur RS, Tousif S, Awasthi V, Sanyal A, Atul PK, Punia P, et al Mesenchymal stem cells play an important role in host protective immune responses against malaria by modulating regulatory T cells. Eur J Immunol. 2013;43(8):2070–7. Epub 2013/05/15. 10.1002/eji.201242882 . 23670483

[36] Soares MB, Lima RS, Rocha LL, Takyia CM, Pontes-de-Carvalho L, de Carvalho AC, et al Transplanted bone marrow cells repair heart tissue and reduce myocarditis in chronic chagasic mice. Am J Pathol. 2004;164(2):441–7. Epub 2004/01/27. 10.1016/s0002-9440(10)63134-3 14742250 PMC1602272

[37] Goes T, Bailao EFLC, Correa CR, Bozzi A, Santos LI, Gomes DA, et al New Developments of RNAi in Paracoccidioides brasiliensis: Prospects for High-Throughput, Genome-Wide, Functional Genomics. Plos Neglected Tropical Diseases. 2014;8(10). 10.1371/journal.pntd.0003173 :000344589000012. PMC4183473 25275433

[38] Macambira SG, Vasconcelos JF, Costa CR, Klein W, Lima RS, Guimaraes P, et al Granulocyte colony-stimulating factor treatment in chronic Chagas disease: preservation and improvement of cardiac structure and function. Faseb j. 2009;23(11):3843–50. Epub 2009/07/18. 10.1096/fj.09-137869 . 19608624

[39] Soares MB, Garcia S, Campos de Carvalho AC, Ribeiro dos Santos R. Cellular therapy in Chagas' disease: potential applications in patients with chronic cardiomyopathy. Regen Med. 2007;2(3):257–64. Epub 2007/05/22. 10.2217/17460751.2.3.257 . 17511562

[40] Soares MB, Santos RR. Current status and perspectives of cell therapy in Chagas disease. Mem Inst Oswaldo Cruz. 2009;104 Suppl 1:325–32. Epub 2009/09/24. 10.1590/s0074-02762009000900043 . 19753492

[41] Soares MB, Lima RS, Souza BS, Vasconcelos JF, Rocha LL, Dos Santos RR, et al Reversion of gene expression alterations in hearts of mice with chronic chagasic cardiomyopathy after transplantation of bone marrow cells. Cell Cycle. 2011;10(9):1448–55. Epub 2011/04/07. 10.4161/cc.10.9.15487 21467843 PMC3117044

[42] Fikry H, Gawad SA, Baher W. Therapeutic Potential of Bone Marrow-Derived Mesenchymal Stem Cells on Experimental Liver Injury Induced by *Schistosoma mansoni*: A Histological Study. Int J Stem Cells. 2016;9(1):96–106. Epub 2016/07/19. 10.15283/ijsc.2016.9.1.96 27426091 PMC4961109

[43] Xu H, Qian H, Zhu W, Zhang X, Yan Y, Mao F, et al Mesenchymal stem cells relieve fibrosis of *Schistosoma japonicum*-induced mouse liver injury. Exp Biol Med (Maywood). 2012;237(5):585–92. Epub 2012/06/09. 10.1258/ebm.2012.011362 . 22678013

[44] Abdel Aziz M, Atta H, Roshdy N, Rashed L, Sabry D, Hassouna A, et al Amelioration of Murine *Schistosoma mansoni* Induced Liver Fibrosis by Mesenchymal Stem Cells. J Stem Cells Regen Med. 2012;8(1):28–34. Epub 2012/01/01. 10.46582/jsrm.0801005 24693190 PMC3908300

[45] Xu HJ, Qian H, Zhu W, Zhang X, Yan YM, Zhang LL, et al [Inhibition of culture supernatant of mesenchymal stem cells on macrophages RAW264.7 activated by soluble egg antigen of *Schistosoma japonicum*]. Zhongguo Ji Sheng Chong Xue Yu Ji Sheng Chong Bing Za Zhi. 2011;29(6):425–30. Epub 2011/12/01. . 24822341

[46] El-Shennawy SF, Abdel Aaty HE, Radwan NA, Abdel-Hameed DM, Alam-Eldin YH, El-Ashkar AM, et al Therapeutic Potential of Mesenchymal Stem Cells on Early and Late Experimental Hepatic Schistosomiasis Model. J Parasitol. 2015;101(5):587–97. Epub 2015/05/27. 10.1645/15-754.1 . 26010300

[47] Gronthos S, Franklin DM, Leddy HA, Robey PG, Storms RW, Gimble JM. Surface protein characterization of human adipose tissue-derived stromal cells. J Cell Physiol. 2001;189(1):54–63. Epub 2001/09/27. 10.1002/jcp.1138 . 11573204

[48] Cahill RA, Wenkert D, Perlman SA, Steele A, Coburn SP, McAlister WH, et al Infantile hypophosphatasia: transplantation therapy trial using bone fragments and cultured osteoblasts. J Clin Endocrinol Metab. 2007;92(8):2923–30. Epub 2007/05/24. 10.1210/jc.2006-2131 . 17519318

[49] Catalog Record: Laboratory methods in histotechnology. 2017.

[50] Pellegrino J, Katz N. Experimental chemotherapy of Schistosomiasis mansoni. Adv Parasitol. 1968;6:233–90. Epub 1968/01/01. 10.1016/s0065-308x(08)60475-3 . 4978052

[51] Oliveira FA, Kusel JR, Ribeiro F, Coelho PM. Responses of the surface membrane and excretory system of Schistosoma mansoni to damage and to treatment with praziquantel and other biomolecules. Parasitology. 2006;132(Pt 3):321–30. Epub 2005/12/02. 10.1017/S0031182005009169 . 16318676

[52] Pellegrino J, Siqueira AF. [A perfusion technic for recovery of *Schistosoma mansoni* from experimentally infected guinea pigs]. Rev Bras Malariol Doencas Trop. 1956;8(4):589–97. Epub 1956/10/01. . 13494879

[53] Lenzi HL, Kimmel E, Schechtman H, Pelajo-Machado M, Romanha WS, Pacheco RG, et al Histoarchitecture of schistosomal granuloma development and involution: morphogenetic and biomechanical approaches. Mem Inst Oswaldo Cruz. 1998;93 Suppl 1:141–51. Epub 1999/01/28. 10.1590/s0074-02761998000700020 . 9921336

[54] Weibel ER, Kistler GS, Scherle WF. Practical stereological methods for morphometric cytology. J Cell Biol. 1966;30(1):23–38. 10.1083/jcb.30.1.23 5338131 PMC2106982

[55] Espíndola MS, Lima LJ, Soares LS, Cacemiro MC, Zambuzi FA, de Souza Gomes M, et al Dysregulated Immune Activation in Second-Line HAART HIV+ Patients Is Similar to That of Untreated Patients. PLoS One. 2015;10(12):e0145261 Epub 2015/12/18. 10.1371/journal.pone.0145261 26684789 PMC4684276

[56] Dominici M, Le Blanc K, Mueller I, Slaper-Cortenbach I, Marini F, Krause D, et al Minimal criteria for defining multipotent mesenchymal stromal cells. The International Society for Cellular Therapy position statement. Cytotherapy. 2006;8(4):315–7. 10.1080/14653240600855905 . 16923606

[57] Sung JH, Yang HM, Park JB, Choi GS, Joh JW, Kwon CH, et al Isolation and characterization of mouse mesenchymal stem cells. Transplant Proc. 2008;40(8):2649–54. Epub 2008/10/22. 10.1016/j.transproceed.2008.08.009 . 18929828

[58] Squillaro T, Peluso G, Galderisi U. Clinical Trials With Mesenchymal Stem Cells: An Update. Cell Transplant. 2016;25(5):829–48. Epub 2015/10/02. 10.3727/096368915X689622 . 26423725

[59] Chen Y, Xiang LX, Shao JZ, Pan RL, Wang YX, Dong XJ, et al Recruitment of endogenous bone marrow mesenchymal stem cells towards injured liver. J Cell Mol Med. 2010;14(6b):1494–508. Epub 2009/09/29. 10.1111/j.1582-4934.2009.00912.x 19780871 PMC3829016

[60] Cho KA, Ju SY, Cho SJ, Jung YJ, Woo SY, Seoh JY, et al Mesenchymal stem cells showed the highest potential for the regeneration of injured liver tissue compared with other subpopulations of the bone marrow. Cell Biol Int. 2009;33(7):772–7. Epub 2009/05/12. 10.1016/j.cellbi.2009.04.023 . 19427913

[61] Kanazawa H, Fujimoto Y, Teratani T, Iwasaki J, Kasahara N, Negishi K, et al Bone marrow-derived mesenchymal stem cells ameliorate hepatic ischemia reperfusion injury in a rat model. PLoS One. 2011;6(4):e19195 Epub 2011/05/12. 10.1371/journal.pone.0019195 21559442 PMC3084802

[62] Martins-Leite P, Gazzinelli G, Alves-Oliveira LF, Gazzinelli A, Malaquias LC, Correa-Oliveira R, et al Effect of chemotherapy with praziquantel on the production of cytokines and morbidity associated with schistosomiasis mansoni. Antimicrob Agents Chemother. 2008;52(8):2780–6. Epub 2008/06/04. 10.1128/AAC.00173-08 18519730 PMC2493106

[63] Dietze R, Prata A. Rate of reversion of hepatosplenic schistosomiasis after specific therapy. Rev Soc Bras Med Trop. 1986;19(2):69–73. Epub 1986/04/01. 10.1590/s0037-86821986000200003 . 3432626

[64] Coelho C, Oliveira FAd, Kusel JR, Fundação Oswaldo Cruz. Centro de Pesquisas René Rachou. Belo Horizonte M, Brasil., Coelho C, Kusel JR, et al. Avaliação do efeito do praziquantel, da oxamniquina e da associação destas drogas sobre o verme adulto de *Schistosoma mansoni* 2005. https://www.arca.fiocruz.br/handle/icict/20772.

[65] Richards F Jr., Sullivan J, Ruiz-Tiben E, Eberhard M, Bishop H. Effect of praziquantel on the eggs of Schistosoma mansoni, with a note on the implications for managing central nervous system schistosomiasis. Ann Trop Med Parasitol. 1989;83(5):465–72. Epub 1989/10/01. 10.1080/00034983.1989.11812373 . 2515814

[66] Alam U. Immunity: The Immune Response to Infectious and Inflammatory Disease. Yale J Biol Med. 2007;80(3):137. PubMed Central PMCID: PMC2248290.

[67] Wynn TA, Cheever AW. Cytokine regulation of granuloma formation in schistosomiasis. Curr Opin Immunol. 1995;7(4):505–11. Epub 1995/08/01. 10.1016/0952-7915(95)80095-6 . 7495514

[68] Jansen BJ, Gilissen C, Roelofs H, Schaap-Oziemlak A, Veltman JA, Raymakers RA, et al Functional differences between mesenchymal stem cell populations are reflected by their transcriptome. Stem Cells Dev. 2010;19(4):481–90. Epub 2009/10/01. 10.1089/scd.2009.0288 . 19788395

[69] Strioga M, Viswanathan S, Darinskas A, Slaby O, Michalek J. Same or not the same? Comparison of adipose tissue-derived versus bone marrow-derived mesenchymal stem and stromal cells. Stem Cells Dev. 2012;21(14):2724–52. Epub 2012/04/04. 10.1089/scd.2011.0722 . 22468918

[70] Hao T, Chen J, Zhi S, Zhang Q, Chen G, Yu F. Comparison of bone marrow-vs. adipose tissue-derived mesenchymal stem cells for attenuating liver fibrosis. Exp Ther Med. 2017;14(6):5956–64. Epub 2017/12/30. 10.3892/etm.2017.5333 29285145 PMC5740792

[71] Lala V, Minter DA. Liver Function Tests. 2018 https://www.ncbi.nlm.nih.gov/books/NBK482489/. 29494096

[72] Larkin BM, Smith PM, Ponichtera HE, Shainheit MG, Rutitzky LI, Stadecker MJ. Induction and regulation of pathogenic Th17 cell responses in schistosomiasis. Semin Immunopathol. 2012;34(6):873–88. Epub 2012/10/26. 10.1007/s00281-012-0341-9 23096253 PMC3690599

