# Supplementary figures and images for: Liver damage in schistosomiasis is reduced by adipose tissue-derived stem cell therapy after praziquantel treatment
Source: PLoS Negl Trop Dis. 2020 Aug 27;14(8):e0008635. doi: 10.1371/journal.pntd.0008635 (PMC7480869; doi:10.1371/journal.pntd.0008635)

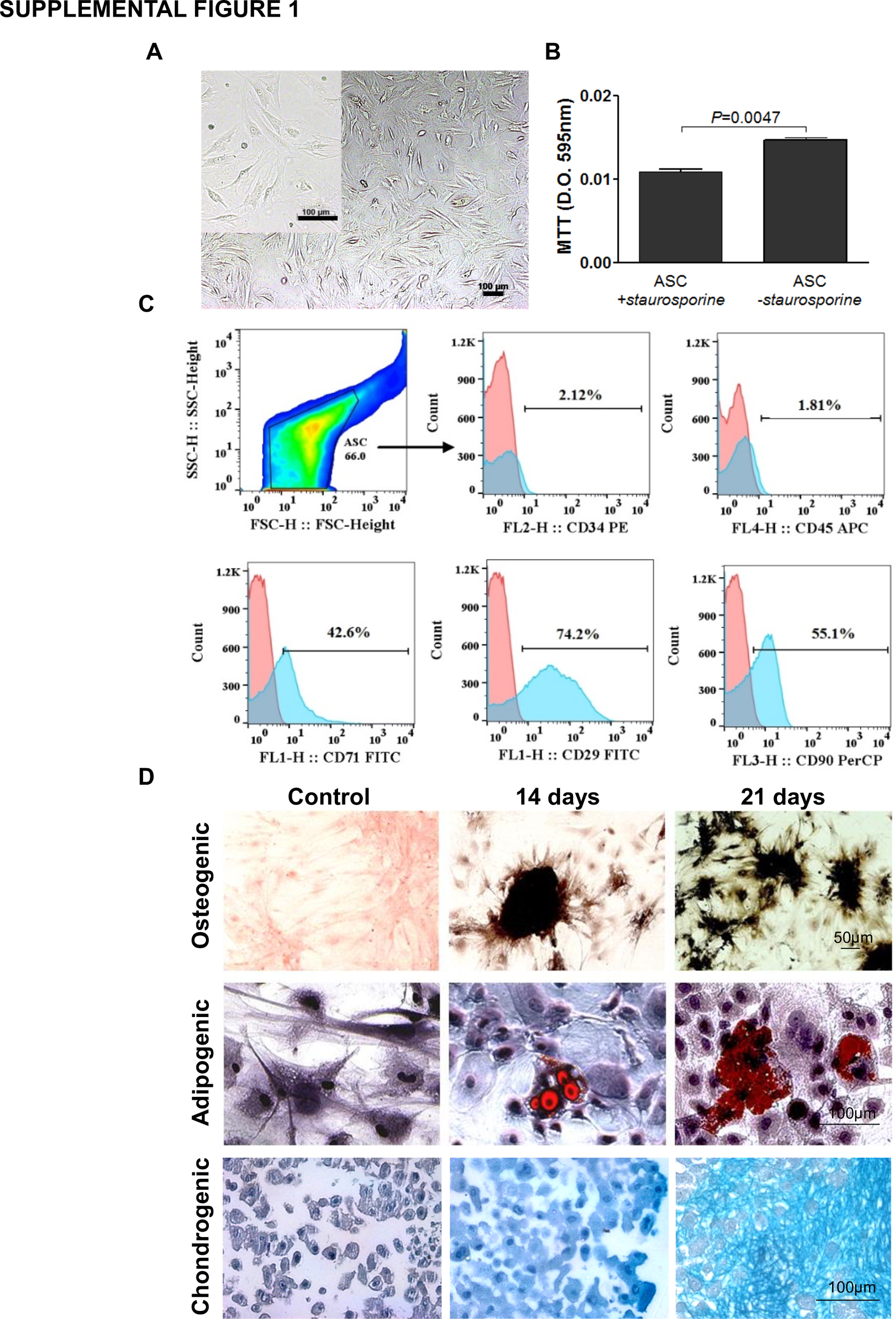

Supplement: S1 Fig — (A) Fibroblast-like morphology of ASC at passage 3 on culture; scale bar 100μm. (B) Viability of ASC evaluated by 3-(4,5-dimethylthiazol-2-yl)-2,5-diphenyltetrazolium bromide (MTT) assay at an optical density (O.D.) at 595nm. Data are presented as mean ± standard error of the mean. (C) Histograms for markers expressed (CD71, CD29, and CD90) or not (CD34 and CD45) by ASC. (D) Osteogenic, adipogenic and chondrogenic multilineage potential of ASC after 14 and 21 days on culture with respective inductors medium. (TIF) [file pntd.0008635.s001.tif]

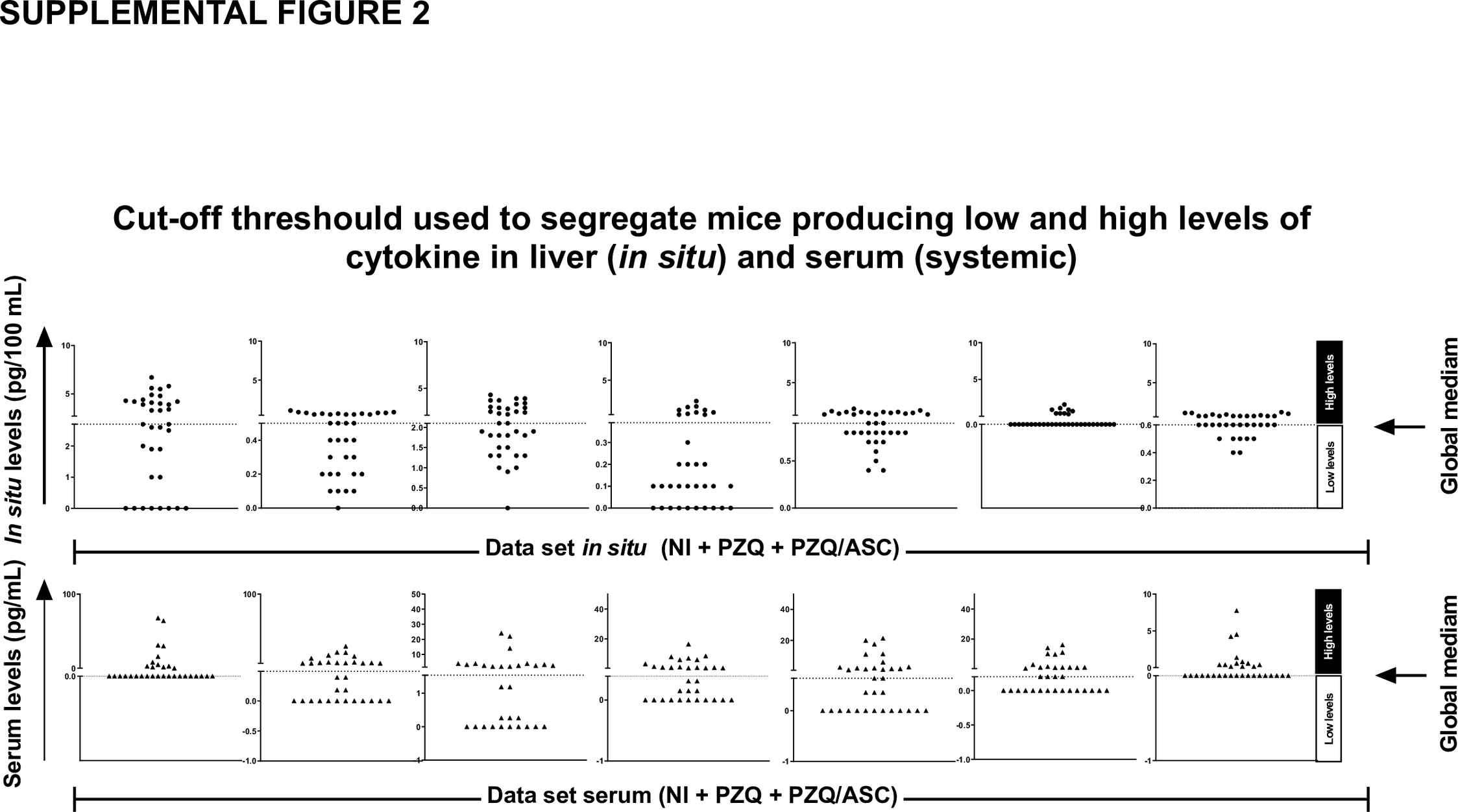

Supplement: S2 Fig — The global median value of all data (NI + PZQ + PZQ/ASC) for each cytokine was calculated and used as the cut-off to classify mice as a “low” (cytokine level under the cut-off) or “high” (cytokine level above the cut-off) producer of a given cytokine. (TIF) [file pntd.0008635.s002.tif]

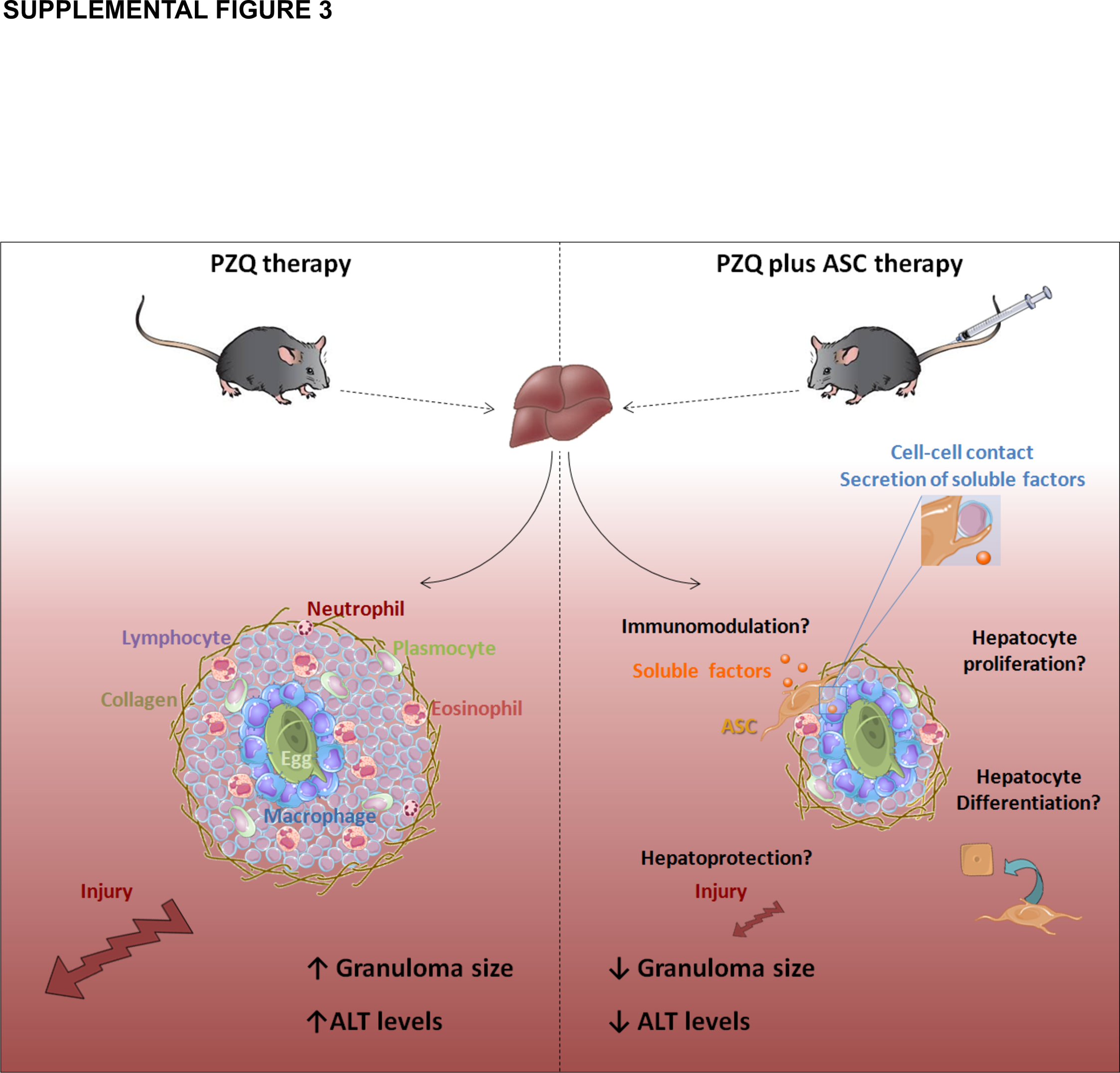

Supplement: S3 Fig — Differences between conventional treatment for schistosomiasis mansoni using PZQ and the proposed treatment of a combination therapy PZQ/ASC. There was a significant decrease in the size of the granulomas in the liver of mice receiving the combination treatment compared to the animals that received only PZQ. In addition, a decrease in serum ALT levels was observed, indicating a reduction of tissue damage. In addition, our results showed that 15 days after injection, ASC were found in the liver. Nonetheless, the mechanics used by ASCs to perform such functions still remain unclear. The complexity of the formation and progression of the granulomatous reaction is one of the reasons why a robust anti-inflammatory therapy has not yet been developed. It may be that a single anti-inflammatory "magic bullet" is simply unable to overcome such complex diseases. Therefore, the presumed effects of ASCs together such as immunomodulation, hepatoprotection, stimulation of hepatic cell proliferation and even differentiation of ASC in hepatocytes may be the answer to the phenomena observed in our study. (TIF) [file pntd.0008635.s003.tif]
